# Peri‐implantitis‐like medication‐related osteonecrosis of the jaw: Clinical considerations and histological evaluation with confocal laser scanning microscope

**DOI:** 10.1111/odi.13873

**Published:** 2021-05-04

**Authors:** Angela Tempesta, Saverio Capodiferro, Rodolfo Mauceri, Dorina Lauritano, Eugenio Maiorano, Gianfranco Favia, Luisa Limongelli

**Affiliations:** ^1^ Department of Interdisciplinary Medicine Complex Operating Unit of Odontostomatology Aldo Moro University of Bari Bari Italy; ^2^ Department of Surgical, Oncological and Oral Sciences University of Palermo Palermo Italy; ^3^ Department of Medicine and Surgery Centre of Neuroscience of Milan University of Milano‐Bicocca Monza Italy; ^4^ Department of Emergency and Organ Transplantation Operating Unit of Pathological Anatomy Aldo Moro University of Bari Bari Italy

**Keywords:** antiangiogenic drugs, antiresorptive drugs, bisphosphonates, medication‐related osteonecrosis of the jaw, peri‐implantitis

## Abstract

**Objective:**

In the recent years, an increasing number of peri‐implant medication‐related osteonecrosis of the jaw (PI‐MRONJ) have been reported in literature, both in oncologic and osteoporotic patients. The aim of this study is to describe 19 cases of patients previously diagnosed as affected by peri‐implantitis, who were treated for PI‐MRONJ, with consideration on clinical and histopathological features.

**Materials and Methods:**

Patients included were affected by postmenopausal osteoporosis and were administered with different antiresorptive drugs. Due to the presence of clinical and radiological signs of peri‐implantitis not healed after non‐surgical periodontal treatment, they were referred to the Complex Operating Unit of Odontostomatology of the University of Bari. Then, after a drug holiday of at least 3 months and cycles of antibiotics, and after other cycles of periodontal treatment, patients underwent the surgical removal of implant fixtures and surrounding bone.

**Results:**

Although the previous diagnosis of peri‐implantitis, the histopathological analysis with both conventional and confocal laser scanner microscopy confirmed the diagnosis of peri‐implantitis‐like MRONJ.

**Conclusion:**

Peri‐implantitis not healed after conventional treatment in patients at risk on MRONJ occurrence should be considered as peri‐implantitis‐like PI‐MRONJ and treated as required in order to get complete healing of the pathological condition, thus avoiding delay in the diagnosis.

## 1 INTRODUCTION

Medication‐related osteonecrosis of the jaw (MRONJ) has been defined by the American Association of Oral and Maxillofacial Surgeons as an exposure of necrotic bone in the oral cavity, or bone that can be probed through an intraoral or extraoral fistula(e) of long lasting duration (within eight weeks after clinical identification) in a patient currently taking or previously treated with antiresorptive or antiangiogenic drugs but never undergone radiotherapy for head and neck neoplasms (Ruggiero et al., 2014).

At the beginning, only bisphosphonates (BPs) administered in patients affected by bone metastases of solid tumours or osteoporosis were considered involved in MRONJ occurrence; nowadays, many other drugs have been described in the English literature as related to MRONJ, such as Denosumab (Favia et al., 2016), Bevacizumab (Antonuzzo et al., 2017), Adalimumab (Cassoni et al., 2016), Infliximab (Favia et al., 2017) and Lenvatinib (Mauceri et al., 2019) prescribed even for rheumatic disorders (rheumatoid arthritis, Crohn's diseases and ulcerative colitis).

Peri‐implantitis is considered the major biological complication associated with the failure of osseointegrated dental implants. It has been defined during the World Workshop on the Classification of Periodontal and Peri‐implant Diseases and Conditions (June 2018, Chicago) as a plaque‐associated pathological condition occurring in tissues around dental implants, characterized by inflammation in the peri‐implant mucosa and loss of supporting bone (Berglundh et al., 2018).

In the recent years, the role of antiresorptive/antiangiogenic drugs administration, and the subsequent possible occurrence of MRONJ, have been investigated as risk factors for implant failure. Because of MRONJ is often related to oral surgical procedures, such as dental extractions (Lam et al., 2007), the role of implant surgery as potential risk factor has been widely debated and there are several guidelines, which remain still controversial (Campisi et al., 2020; Ruggiero et al., 2009); in the recent years, an increasing number of peri‐implant MRONJ (PI‐MRONJ) have been reported in literature both in oncologic and osteoporotic patients (Bedogni et al., 2010; Favia et al., 2011; Kwon et al., 2012; Lazarovici et al., 2010). PI‐MRONJ have been classified as *implant surgery‐triggered* (onset within six months after implant placement and strictly related to the surgical procedure or a failure in osseointegration process) and *non‐implant surgery‐triggered* (onset more than six months after implant placement, or drug therapy started after implant placement and osseointegration) (Kwon et al., 2012).

The possible association between MRONJ and peri‐implantitis has rarely been investigated in the current English literature (Troeltzsch et al., 2016). The clinico‐radiological presence of peri‐implantitis‐like aspects in patients at risk of MRONJ development causes diagnostic problems between the diagnosis of simple peri‐implantitis or that of PI‐MRONJ.

The aim of this study is to report on the cases of 19 osteoporotic patients undergoing antiresorptive drugs with a previous diagnosis of peri‐implantitis affected by PI‐MRONJ, describing their salient clinical and histopathological features by using both conventional and confocal laser scanning microscopy (CLSM).

## 2 MATERIALS AND METHODS

This study was carried out in accordance with the principles of the Declaration of Helsinki and was approved by our Institutional Ethical Committee (study number 3,211, Prot. 201/C.E.‐30/01/2008); the patients released the informed consent on diagnostic and therapeutic procedures and possible use of the biological samples for research purposes.

All the enrolled patients were sent to the Complex Operating Unit of Odontostomatology of Aldo Moro University of Bari from 2010 to 2019 by their private dental practitioner, due to the presence of peri‐implantitis not healed after non‐surgical treatment. The inclusion criteria were as follows:

1. Patients affected by postmenopausal osteoporosis who were administered antiresorptive drugs;
2. Presence of osseointegrated dental implants placed successfully more than 6 months before the beginning of antiresorptive drugs administration;
3. Diagnosis of peri‐implantitis around one or more dental implants, not healed after non‐surgical peri‐implant treatment.

During general anamnesis, the following clinical data were collected: age, patient‐related parameters (data on osteoporosis, presence of comorbidities), type, dose and duration of antiresorptive therapy, and information about dental implants (number, position, time of implant insertion, time between implant placement and antiresorptive therapy).

**FIGURE 1:**
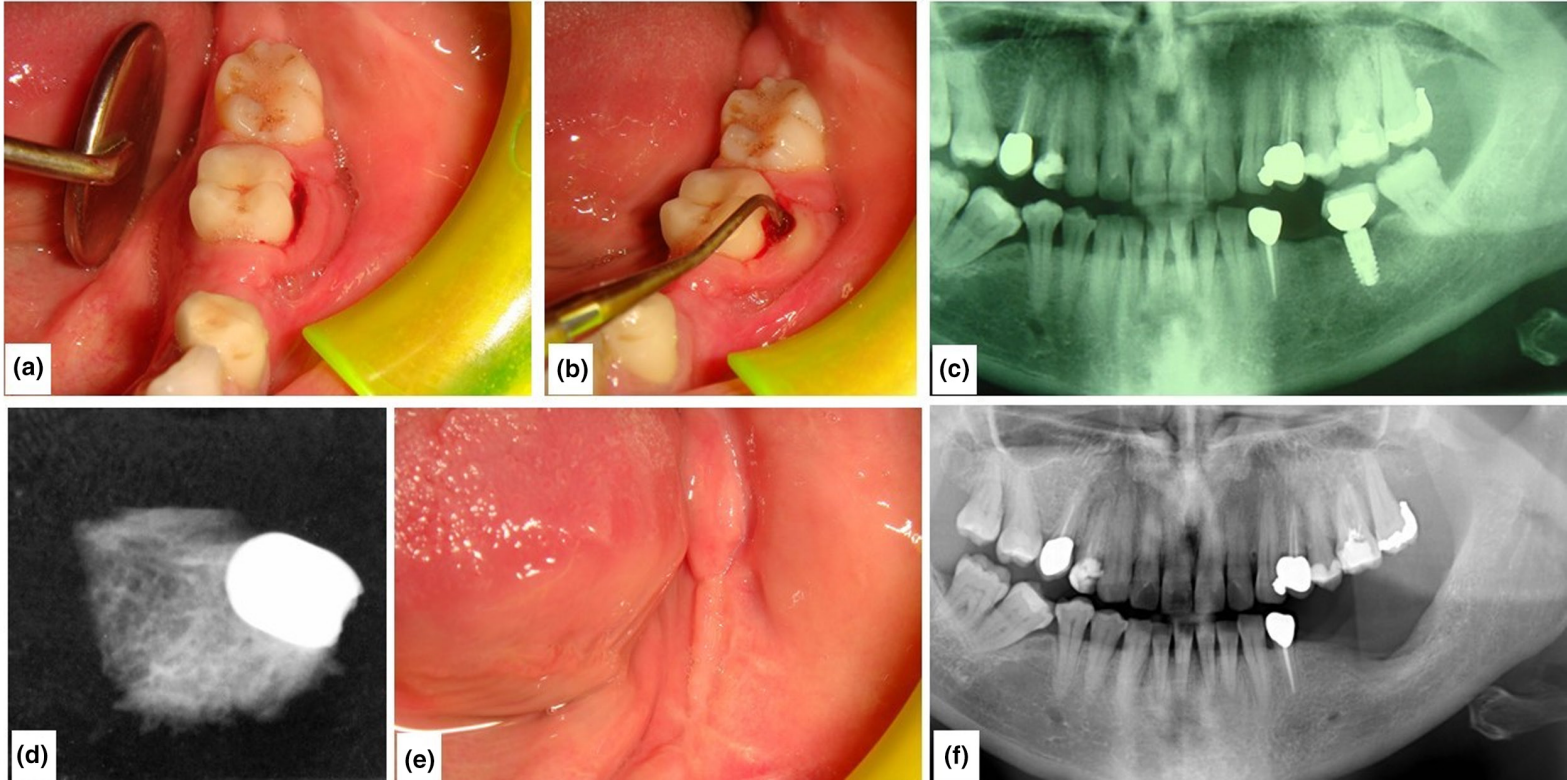
Clinical and radiological images of a patient before and after surgery

All patients underwent the same diagnostic‐therapeutic protocol consisting in:

- Clinical examination: evaluation of signs of peri‐implantitis (Figure 1a,b) according with diagnostic criteria reported in *The new classification of periodontal and peri‐implant diseases and conditions—Consensus from world workshop in Chicago,* June 2018 (bleeding and/or suppuration on gentle probing; probing depths of >6 mm and bone levels >3 mm apical of the most coronal portion of the intra‐osseous part of the implant; bone loss beyond crestal bone‐level changes resulting from initial bone remodelling)
- Radiological assessment: intraoral Rx, panoramic radiogram (Figure 1c) and multi‐slices spiral computed tomography (CT) with three‐dimensional reconstruction.

Considering the diagnosis of peri‐implantitis for the patients treated before 2018, a reclassification of the peri‐implant pathologic condition was performed by a retrospective analysis of periodontal charts saved in archives.

Then, considering the risk of MRONJ occurrence for these patients, in accordance with patients' general physicians, antiresorptive drugs administration was suspended and treatment of peri‐implantitis was made at controlling infection by the elimination of the biofilm from the implant surface through a non‐surgical approach. Implant fixtures were cleaned by instruments softer than titanium, such as plastic scaling instruments and ultrasonic scalers with a non‐metallic tip. Submucosal treatment with diode laser and antimicrobial application (metronidazole 40% gel) into peri‐implant pocket was also performed. Before each session, the persistence of peri‐implantitis was defined by the evaluation of the probing depth values and presence of gingival bleeding.

If signs of peri‐implant pathologic condition did not heal after 30 days and at least five sessions of such periodontal treatment, considering that peri‐implantitis itself can be considered a risk factor for MRONJ occurrence, surgical treatment was planned. Before the surgical treatment, that cannot be performed less than 3 months after antiresorptive drugs discontinuation, combined antibiotic therapy (consisting of a combination of ceftriaxone 1 g via intra‐muscle once a day and metronidazole 500 mg twice a day) for 8 days, repeated three times with a drug‐free period of 10 days, was prescribed.

During surgery, first step consisted in removing implant fixtures with surrounding soft tissues; due to the presence of not vascularized necrotic greenish osseous tissues, surrounding bone was also removed (Figure 1d) and bone core was performed for histopathological diagnosis. Then, osteoplasty with both rotative and piezosurgical devices of the residual resection margins was executed, and a medical device made of hyaluronic acid and amino acids (glycine, leucine, lysine and proline) was put into the bone defect to accelerate the wound healing.

All the surgical samples were promptly fixed in 10% neutral buffered formalin and sent for the histopathological examination. Samples were decalcified in formic acid (5% in distilled water) for 24 hr, embedded in paraffin, sectioned and stained with two different methods: haematoxylin–eosin for traditional microscopy and picrosirius red for CLSM. The histopathological analysis was performed using Nikon Eclipse E600 microscope (Nikon Corporation), equipped with Argon‐ion and Helio‐Neon lasers (488 and 543 nm wavelengths, respectively), thus allowing both optical and confocal laser scanning examination. The Nikon EZ C1 software (Nikon Corporation, ver. 2.10) was used for the bi‐ and tri‐dimensional image processing.

Patients underwent clinical follow‐up, respectively, after 2, 7, 15 and 30 days, and panoramic radiogram was repeatedly performed once a month (Figure 1e,f).

## 3 RESULTS

Overall, 19 female patients (average age 64.9 ± 8.3 y.o.,) were included in the current study. They all had diagnosis of postmenopausal osteoporosis treated with antiresorptive drugs.

Alendronate was administered to six of them, Denosumab to 5, Risedronate to 4, Clodronate to 2 and Ibandronate to 2. Implant placement and duration of antiresorptive therapy were, respectively, on average of 45 months and 27 months before PI‐MRONJ occurrence. Totally, patients showed 37 dental implants presenting signs of peri‐implantitis; of them, 14 were placed on the anterior lower jaw, 10 on the posterior lower jaw, 7 on the posterior upper jaw and 6 on the anterior upper jaw. Moreover, no signs of periodontitis or presence of bone exposure at other locations were evaluated. All patients’ data are reported in Table 1.

**TABLE 1:** Patients’ data

Patient	Gender	Age	Antiresorptive drug	n implants	Site	Tobacco habits	Other comorbidities	Other drugs
1	F	79	Risedronate	1	anterior lower jaw	No	Atrial fibrillation	Anticoagulant
2	F	61	Alendronate	1	anterior upper jaw	No	Hypertension	Beta‐blockers
3	F	78	Denosumab	1	anterior lower jaw	No	Diabet	Metformin
4	F	59	Denosumab	1	posterior lower jaw	Yes	None	‐
5	F	62	Alendronate	1	anterior upper jaw	No	None	‐
6	F	67	Risedronate	1	posterior lower jaw	Yes	Hypothyroidism	Levothyroxine
7	F	54	Risedronate	1	anterior lower jaw	Yes	None	‐
8	F	69	Denosumab	1	posterior lower jaw	No	None	‐
9	F	56	Alendronate	2	anterior lower jaw	No	Gastroesophageal reflux disease	Pantoprazole
10	F	78	Alendronate	2	anterior upper jaw	Yes	Diabet	Metformin
11	F	71	Clodronate	2	anterior lower jaw	No	None	‐
12	F	62	Alendronate	2	posterior upper jaw	Yes	None	‐
13	F	68	Denosumab	2	posterior lower jaw	No	Diabet	Metformin
14	F	70	Ibandronate	2	anterior lower jaw	No	Hypothyroidism	Levothyroxine
15	F	69	Clodronate	2	posterior upper jaw	No	None	‐
16	F	67	Denosumab	3	anterior lower jaw	Yes	None	‐
17	F	53	Ibandronate	3	posterior lower jaw	Yes	Hypertension	Beta‐blockers
18	F	57	Risedronate	2	posterior lower jaw	No	None	‐
				2	anterior lower jaw	No		
19	F	54	Alendronate	5	posterior+anterior upper jaw	No	None	‐

Considering signs of peri‐implantitis, all implants showed bleeding on probing and increasing probing depth (the average probing depth was 7 ± 1.2 mm), while suppuration was detected around 23 implants, implant mobility was found in 13 cases. At radiological examination, the presence of bone defects typical of peri‐implantitis was also evaluated.

After the conventional non‐surgical treatment of peri‐implantitis, although an improvement of signs was detected in some cases, no one case of complete healing was evaluated. For such reason, all patients underwent the surgical removal of implants and surrounding tissues. All treated lesions healed without signs of recurrence after a mean clinical and radiological follow‐up of 20 months.

**FIGURE 2:**
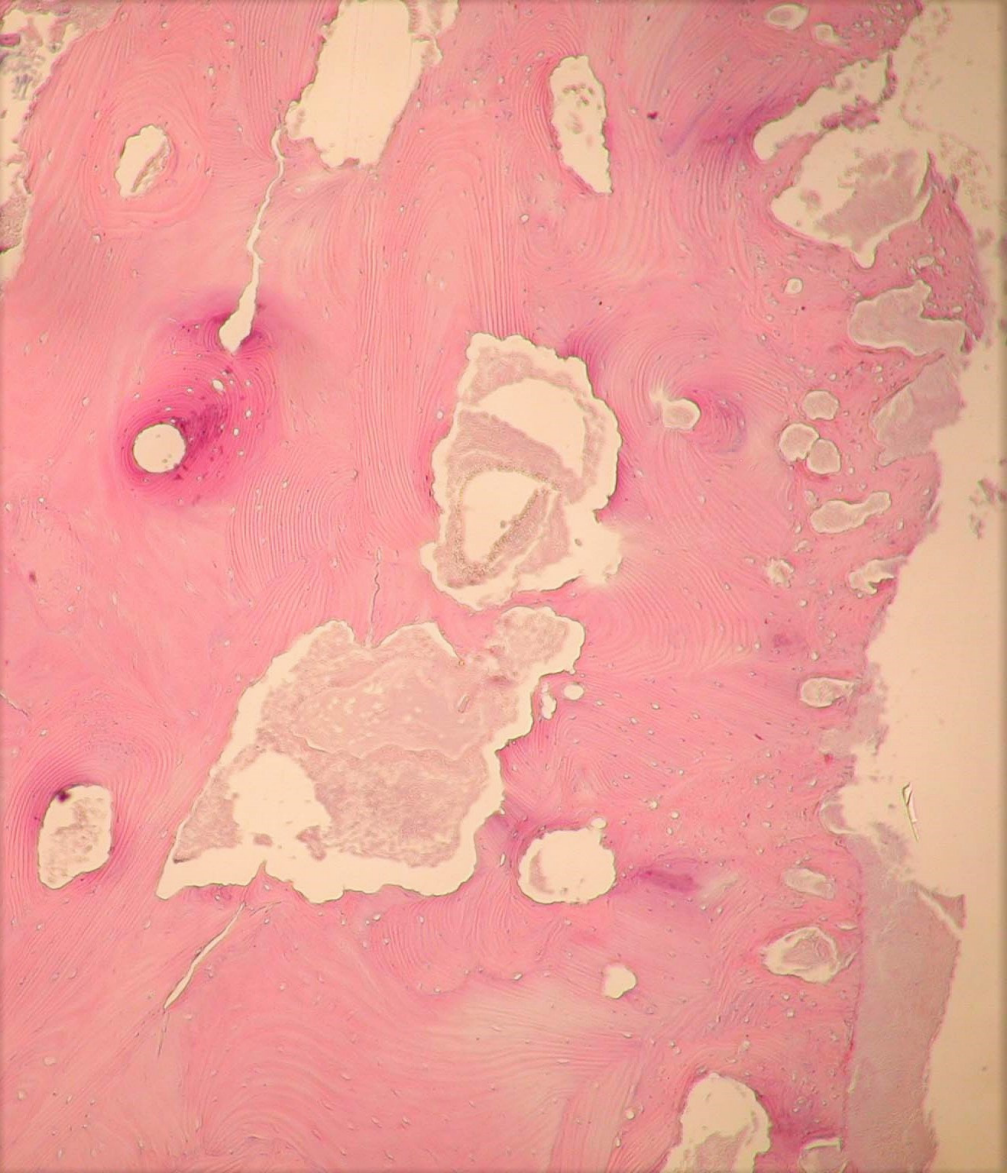
Histopathological aspects of bone–implant fixtures interface

**FIGURE 3:**
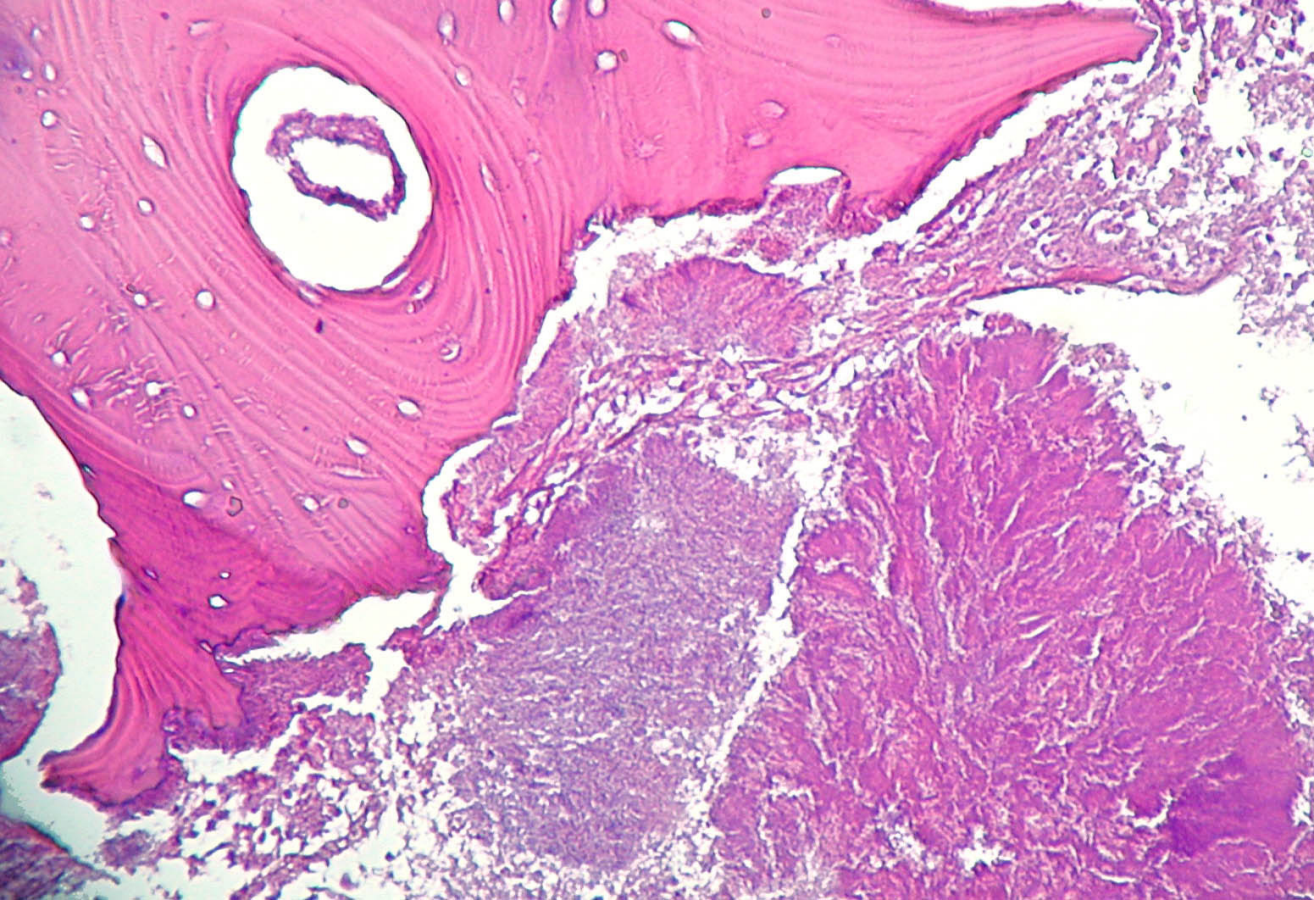
Necrotic bone with bacterial colonies

**FIGURE 4:**
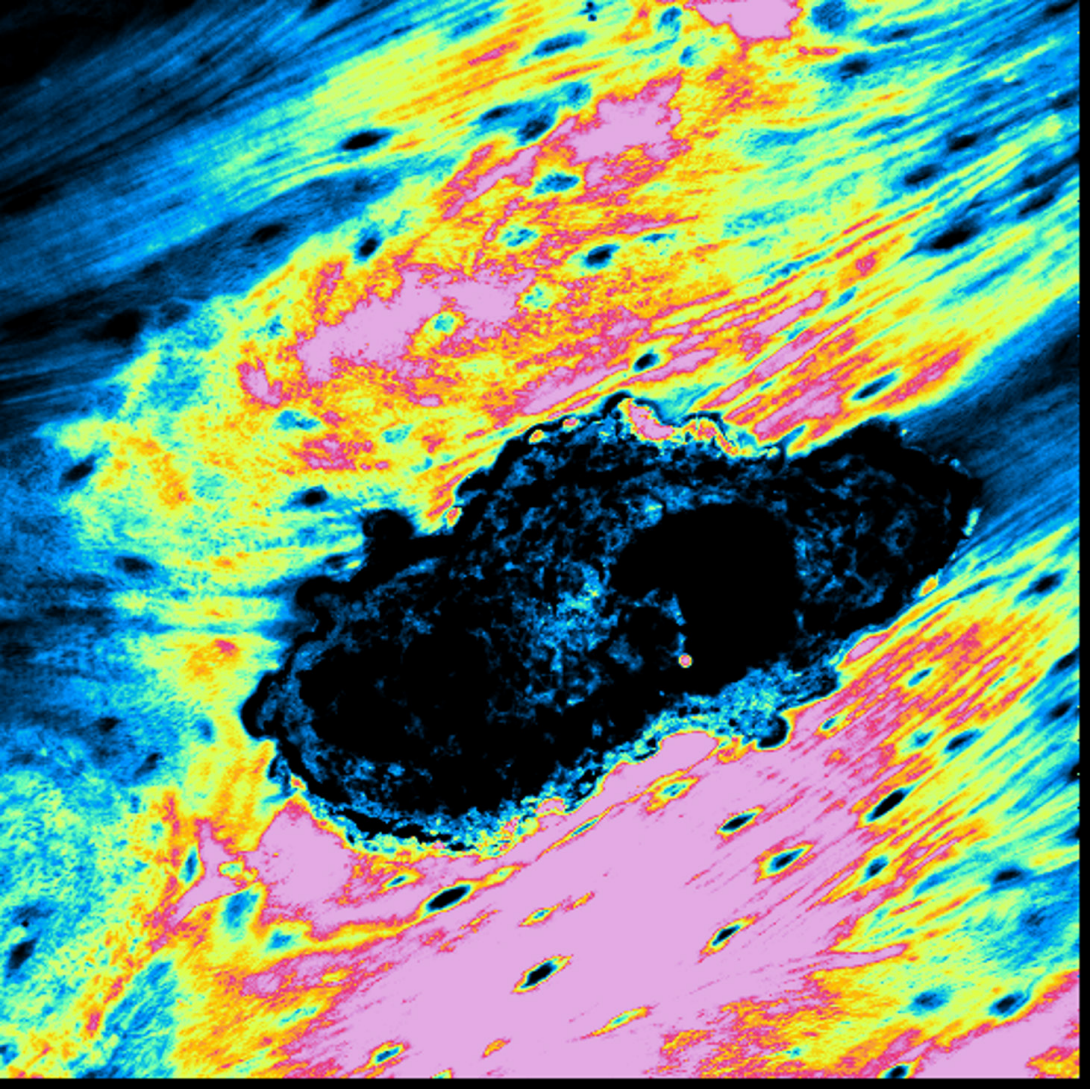
CLSM showed hypercalcified osteonic structures, with rare and empty osteocytic lacunae

The conventional histopathological examination of the surgical samples revealed the presence of large areas of bone resorption at implant fixtures–bone interface (Figure 2), which certainly caused radiological radiolucency around implants, with areas of active acute and chronic phlogosis, characterized by polymorphonuclear phagocytes, plasma cells, monocytes and lymphocytes (Figure 3). Moreover, peculiar features of MRONJ were observed:

- irregular shaped and dilated Haversian channels filled of dense inflammatory infiltrate with prominent granulocytic cells, and with active resorption of the channel walls, and absence of vascular structures (Figure 2);
- presence of avascular bone necrosis in the bone surrounding the Haversian channels (Figure 2);
- macro‐lamellar bone with large concentric hypercalcified osteonic structures, containing rare, clear and empty osteocytic lacunae (osteocytic necrosis) (Figures 2 and 4);
- presence of basophilic bacterial colonies interspersed within the bony necrotic debris and the peri‐implant soft tissues, and fulfilling the Haversian channels closed the implant site (Figure 3).

**FIGURE 5:**
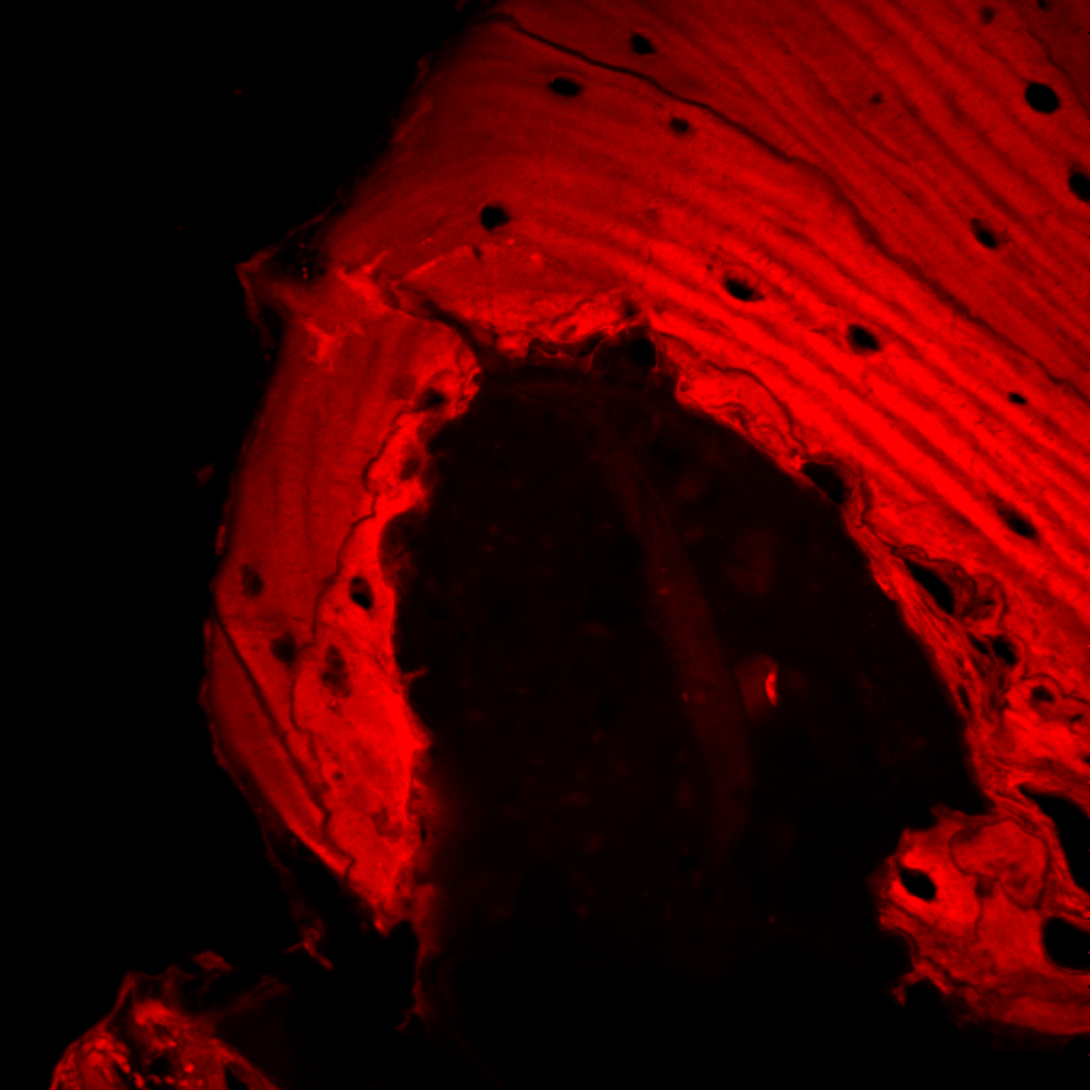
Internal osteoclastic resorption of Haversian channels at CLSM with 3D reconstruction

At CLSM analysis with the 3D reconstruction with red fluorescence (Figure 5), it underlined the intense internal osteoclastic resorption of the Haversian channels, showing multiple Howship's lacunae especially. So, the final diagnosis of peri‐implantitis‐like PI‐MRONJ was made and, because implants were placed more than 6 months before the beginning of the antiresorptive and/or antiangiogenic treatment, all PI‐MRONJ were classified as “non‐surgery‐triggered” and were considered spontaneous.

## 4 DISCUSSION

After the first case described by Marx (Marx, 2003), cases of MRONJ have been reported increasingly and emerged as one of the major adverse side effects of antiresorptive and antiangiogenic therapy (Kwon et al., 2012). The pathogenesis seems to be related to a defect in jawbone physiologic remodelling, due to the strong inhibition of osteoclast activity with the following alteration of the normal bone turn over after surgical procedure or after local microdamage from the normal mechanical loading (Ruggiero & Drew, 2007; Santarelli et al., 2014). Recently, PI‐MRONJ has been described (Kwon et al., 2012), but, to date, among all the procedures of oral and maxillofacial surgery, it remains unclear if implant surgery represents a true risk factor for MRONJ development.

Some authors considered BPs treatment as an absolute contraindication to oral implants, and more precisely both insertion of dental implants (*implant surgery‐triggered* MRONJ) and the presence itself of a fixture (*non‐implant surgery‐triggered* MRONJ) represent a high risk factor for MRONJ occurrence, the latter appearing as a permanent risk factor for the development MRONJ (Scully et al., 2006).

In contrast, other authors, such as Bell et al. Bell & Bell, (2008), Grant et al. Grant et al. (2008) and Koka et al. Koka et al. (2010), report on the lack of evidence of an increased risk of MRONJ in patients taking oral BPs and receiving dental implants, suggesting that implant therapy in BP users was a “safe and predictable procedure that did not require a drug holiday.”

On the contrary, in the same year, Goss et al. Goss et al. (2010) sustained that a “*certain amount of risk*” related to implant surgery for patients taking oral BPs exists, both for *implant surgery‐triggered* and *non‐implant surgery‐triggered* MRONJ. Similar results have been reported by Jacobsen et al. Jacobsen et al. (2013); Bedogni et al. Bedogni et al. (2010) concluded that despite the low risk of MRONJ occurrence after implant surgery in oral BPs users, the success of dental implants remains uncertain. Therefore, all patients should be accurately informed about the potential risk of implant failure and PI‐MRONJ development both in the short and long term, and great attention should be paid to the long term maintenance of a good oral hygiene.

The few published histological studies on PI‐MRONJ described three general patterns of bone involvement: (a) bone necrosis around the fixture, (b) extensive osteolysis around the fixture with or without sequestrum (peri‐implantitis‐like type) and (c) sequestration of bone with a preserved direct implant–bone contact (en‐block type) (Kwon et al., 2012). In addition, these findings could also co‐exist within the same lesion, the latter remaining strictly related to the severity both of bone destruction and infection associated (Favia et al., 2011).

The data available in the literature demonstrate that the occurrence of MRONJ in patients undergoing antiresorptive and antiangiogenic therapy and needing implant‐supported prosthetic rehabilitation or presenting dental implants is still an open and debated question.

Considering non‐surgery‐triggered PI‐MRONJ, the role of peri‐implantitis as risk factor has been rarely investigated. Troeltzsch et al., Troeltzsch et al. (2016) in 2016, reported that signs of peri‐implantitis appeared to be associated with the occurrence of PI‐MRONJ in 39% of their case study, comprehending 117 dental implants in 34 patients.

In the current study, 19 patients were included. All of them were previously treated by their dental practitioner for peri‐implantitis without healing of treated sites and, then, were sent to our attention. Considering patients’ anamnesis and previous administration of antiresorptive drugs, we considered the hypothesis that peri‐implantitis could be a sign of PI‐MRONJ. Consequently, conventional treatment of peri‐implantitis was performed again during antiresorptive drug suspension, and all not healed lesions were surgically treated.

Both conventional and CLSM histopathological analyses of surgical specimens highlighted that although peri‐implant tissues showed bone resorption and inflammation typical of peri‐implantitis, the surrounding bone was characterized by the histopathological features of MRONJ (Favia et al., 2009), thus confirming the diagnosis of PI‐MRONJ. Because implant placement was successfully performed more than 6 months before antiresorptive drugs administration, all PI‐MRONJ were considered as spontaneous.

This data demonstrate that typical signs of peri‐implantitis could be uncommon signs PI‐MRONJ in patients taking antiresorptive/antiangiogenic drugs, even if usual signs of MRONJ, as bone exposure or intra/extraoral fistula are not present. For such reasons, the differential diagnosis between peri‐implantits and PI‐MRONJ is not always easy, thus producing delayed diagnosis. Consequently, it would be advisable to treat all peri‐implantitis not healed after treatment as PI‐MRONJ by surgical approach. Probably, the duration of drugs administration could be related to a higher onset of PI‐MRONJ, thus considering that cellular bone turnover is progressively and increasingly modified in close relationship to the duration of the therapy (Franco et al., 2014). Many other adjunctive factors should be considered in such patients, as the characteristics of the prosthetic rehabilitation, including the precision of implant‐abutment and/or abutment‐crown connection, could influence the functional loading and the maintenance of a good domestic oral hygiene. Moreover, it must not be underestimate the possibility that these cases of non‐implant surgery‐triggered PI‐MRONJ, an initial peri‐implantitis could be the local causing factor for ONJ occurrence, thus suggesting the importance of prevention of peri‐implantitis in these patients.

On the basis of data reported in the international literature and in addiction of the results of the current study, the presence of dental implants should be always considered as an at‐risk situation, therefore needing accurate attention and prevention. Peri‐implantitis not healed after conventional non‐surgical treatment in patients taking antiresorptive/antiangiogenic drugs has to be considered as peri‐implantitis‐like PI‐MRONJ and treated as required in order to get complete healing of the pathological condition. So, according to author's experience, the removal of pathologic implants with surrounding bone is always mandatory in these patients considering the stage of PI‐MRONJ diagnosed, as described in this study. Peri‐implantitis itself could be the trigger factor for PI‐MRONJ, so prevention is primary in these patients.

We retained that further review studies are needed to assess general guidelines for PI‐MRONJ prevention, early diagnosis and management.

## CONFLICT OF INTEREST

The authors declare no conflict of interest.

## AUTHOR CONTRIBUTIONS

**Angela Tempesta:** Conceptualization; Data curation; Investigation; Methodology; Validation; Writing‐original draft; Writing‐review & editing. **saverio capodiferro:** Investigation; Methodology; Writing‐original draft; Writing‐review & editing. **Rodolfo** **Mauceri:** Validation; Visualization; Writing‐original draft; Writing‐review & editing. **Dorina Lauritano:** Supervision; Validation; Visualization; Writing‐review & editing. **Eugenio** **Maiorano:** Supervision; Validation; Writing‐review & editing. **Gianfranco** **Favia:** Conceptualization; Data curation; Investigation; Supervision; Validation; Visualization; Writing‐review & editing. **Luisa** **Limongelli:** Data curation; Investigation; Methodology; Supervision; Writing‐original draft; Writing‐review & editing.

### PEER REVIEW

The peer review history for this article is available at https://publons.com/publon/10.1111/odi.13873.
